# Parenteral Nanoemulsion for Optimized Delivery of GL-II-73 to the Brain—Comparative In Vitro Blood–Brain Barrier and In Vivo Neuropharmacokinetic Evaluation

**DOI:** 10.3390/pharmaceutics17030354

**Published:** 2025-03-10

**Authors:** Kristina Jezdić, Jelena Đoković, Ivan Jančić, Tanja Ilić, Biljana Bufan, Bojan Marković, Jana Ivanović, Tijana Stanković, Nebojša D. Cekić, Vassiliki Papadimitriou, Dishary Sharmin, Prithu Mondal, James M. Cook, Snežana D. Savić, Miroslav M. Savić

**Affiliations:** 1 Department of Pharmacology, Faculty of Pharmacy, University of Belgrade, 11221 Belgrade, Serbia; kristina.mirkovic@pharmacy.bg.ac.rs (K.J.); jana.ivanovic@pharmacy.bg.ac.rs (J.I.); miroslav@pharmacy.bg.ac.rs (M.M.S.); 2 Department of Pharmaceutical Technology and Cosmetology, Faculty of Pharmacy, University of Belgrade, 11221 Belgrade, Serbia; jelena.djokovic@pharmacy.bg.ac.rs (J.Đ.); tanja.ilic@pharmacy.bg.ac.rs (T.I.); tijana.stankovic@pharmacy.bg.ac.rs (T.S.); snezana.savic@pharmacy.bg.ac.rs (S.D.S.); 3 Department of Microbiology and Immunology, Faculty of Pharmacy, University of Belgrade, 11221 Belgrade, Serbia; biljana.bufan@pharmacy.bg.ac.rs; 4 Department of Pharmaceutical Chemistry, Faculty of Pharmacy, University of Belgrade, 11221 Belgrade, Serbia; bojan.markovic@pharmacy.bg.ac.rs; 5 Faculty of Technology, University of Niš, 16000 Leskovac, Serbia; ncekic@tf.ni.ac.rs; 6 DCP Hemigal, 16000 Leskovac, Serbia; 7 Institute of Chemical Biology, National Hellenic Research Foundation, 11635 Athens, Greece; vpapa@eie.gr; 8 Department of Chemistry and Biochemistry, Milwaukee Institute for Drug Discovery, University of Wisconsin-Milwaukee, Milwaukee, WI 53211, USA; dsharmin@uwm.edu (D.S.); pmondal@uwm.edu (P.M.); capncook@uwm.edu (J.M.C.)

**Keywords:** blood–brain barrier, α5GABA_A_ receptors, neuropharmacokinetics, brain targeting, GL-II-73

## Abstract

**Background/Objectives**: GL-II-73 is a positive allosteric modulator that is selective for α5GABA_A_ receptors and has physicochemical properties that favor nanocarrier formulations when parenteral delivery to the central nervous system is desired. Our aim was to develop an optimized nanoemulsion containing GL-II-73 and subsequently test whether this would improve permeation across the blood–brain barrier (BBB) and availability in the brain. **Methods**: The nanoemulsions were formulated and subjected to detailed physiochemical characterization. The optimized formulation was tested in comparison to a solution of GL-II-73 in the appropriate solvent in an in vitro model of the blood–brain barrier based on human induced pluripotent stem cell-derived microvascular endothelial cells, astrocytes, and pericytes. Plasma and brain exposure to GL-II-73 and its metabolite MP-III-022 was investigated in an in vivo neuropharmacokinetic study in rats exposed to the selected nanoemulsion and the conventional solution formulation. **Results**: The selected biocompatible nanoemulsion exhibited satisfactory physicochemical properties for parenteral administration, with a Z-ave of 122.0 ± 1.5, PDI of 0.123 ± 0.009 and zeta potential of −40.7 ± 1.5, pH of 5.16 ± 0.04, and adequate stability after one year of storage, and allowed the localization of GL-II-73 in the stabilization layer. The permeability of GL-II-73 through the BBB was twice as high with the selected nanoemulsion as with the solution. The availability of GL-II-73 and MP-III-022 (also a positive allosteric modulator selective for α5GABA_A_ receptors) in the brain was 24% and 61% higher, respectively, after intraperitoneal administration of the nanoemulsion compared to the solution; the former increase was statistically significant. **Conclusions**: The increased permeability in vitro proved to be a good predictor for the improved availability of GL-II-73 in brain tissue in vivo from the formulation obtained by encapsulation in a nanoemulsion. The putative additive effect of the parent molecule and its metabolite MP-III-022 could lead to enhanced and/or prolonged modulation of α5GABA_A_ receptors in the brain.

## 1. Introduction

Despite significant advances, the treatment of neurodegenerative, cerebrovascular, and psychiatric diseases and central nervous system (CNS) infections remains a major challenge [1]. Part of this difficulty arises from the restrictive nature of the blood–brain barrier (BBB), which limits the efficacy of an estimated 98% of existing drugs for brain diseases by preventing them from reaching the pathological areas in optimal amounts [2]. An illustrative example of the active search for additional approaches to bypass the BBB is the effort to utilize the recently discovered glymphatic system as a paravascular pathway, similar to the lymphatic system, for more effective brain drug delivery [3].

Current trends in drug discovery for brain diseases favor the development of lipophilic drugs with moderate to poor water solubility to improve their passage through the BBB [4]. Various lipid nanocarriers (liposomes, nanoemulsions, solid lipid nanoparticles, and nanostructured lipid carriers) have been used for the delivery of poorly soluble molecules, supported by innovative excipients and ligands for the targeted delivery and controlled release of drugs [5].

**Figure 1:**
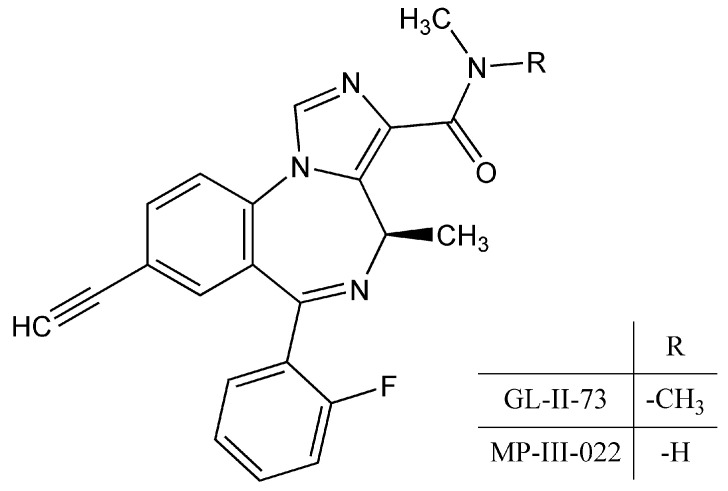
Chemical structures of GL-II-73 and its mono-demethyl metabolite MP-III-022.

Ligands of the benzodiazepine chemotype represent one of the most promising niches for the discovery of novel CNS drugs, considering the diversity of their current and potential clinical applications and the opportunities offered by selective activity at different GABA_A_ receptor subtypes, which can achieve an optimized balance between the benefit and safety of future drugs [6]. GL-II-73 (Figure 1) is a newer imidazobenzodiazepine derivative that has been described as a positive allosteric modulator at the benzodiazepine binding site selective for GABA_A_ receptors containing the α5 subunit (α5GABA_A_ receptors) [7]. It has shown procognitive and rapid antidepressant effects in rodents, with particularly pronounced activity at the level of the hippocampus [7,8,9].

GL-II-73 is a lipid-soluble ligand with moderate water solubility. While most previous studies in rodents were performed after intraperitoneal administration of a conventional formulation of GL-II-73 (e.g., refs. [7,8,9]), it is legitimate to hypothesize that an appropriate nanostructured formulation could improve the neuropharmacokinetic performances of GL-II-73. As an example, the study by Tan et al. (2015) on the nanoemulsion-based parenteral delivery system of carbamazepine showed that nanoemulsions improve the bioavailability of the drug and its ability to cross the BBB [10]. Nanoemulsions are oil-in-water systems stabilized by surfactants, where the solubility of the drug in oils is crucial for the selection of components [11]. They are particularly suitable for parenteral administration given their association with reduced pain during application compared to the other nonlipidic formulations [12].

Improving the delivery of GL-II-73 to the brain could further enhance its therapeutic potential. To address the issue of BBB permeability and CNS availability, our research focused on developing a customized formulation of a nanoemulsion containing GL-II-73 and evaluating its permeability using an in vitro BBB model as well as its exposure in the brain by conducting a neuropharmacokinetic study. In addition, it would be interesting to compare not only the pharmacokinetic profiles of GL-II-73 from a nanostructured and a conventional formulation, but also the profiles of its putative major metabolite MP-III-022 (Figure 1), which presumably results from the demethylation of GL-II-73. Importantly, MP-III-022 is also a selective α5-GABA_A_ receptor ligand that has shown pharmacological efficacy at relatively low doses in rodent models of various psychiatric disorders, including autism [13], schizophrenia [14], and cognitive impairment [15]. In this sense, pharmacologically relevant concentrations of MP-III-022 in the brain could increase the overall capacity of GL-II-73 to potentiate α5GABA_A_ receptors.

Therefore, our aim was to develop an optimized nanoemulsion containing GL-II-73 and subsequently to investigate the pharmacokinetic and neuropharmacokinetic parameters of GL-II-73 and its metabolite MP-III-022 in rat plasma and brain, respectively. In addition, a parallel interpretation of the in vitro BBB and in vivo neuropharmacokinetic behavior would help to assess the extent to which a formulated nanoemulsion improves the targeted availability of GL-II-73 in the brain compared to the conventional formulation. Thus, the novelty of this work lies in the parallel use of state-of-the-art tools for in vitro (human cell-based tricellular BBB model) and in vivo (neuropharmacokinetic study in experimental animals) assessment of the actual utility of a nanodelivery platform tailored to the physicochemical properties of a CNS-active ligand with potential clinical use.

## 2. Materials and Methods

### 2.1. Preparation of Nanoemulsions

#### 2.1.1. Materials

GL-II-73 was synthesized by the research group of Dr. James M. Cook (Department of Chemistry and Biochemistry, University of Wisconsin-Milwaukee, Milwaukee, WI, USA).

Buthylhidroxytoluen (BHT) and polysorbate 80 were purchased from Sigma-Aldrich Co. (St. Louis, MO, USA), while medium-chain triglycerides (MCTs) were obtained from Fagron GmbH & KG (Barsbüttel, Germany). Glycerol was provided by Merck KGaA (Darmstadt, Germany). Soybean oil (Lipoid Purified Soybean Oil 700), soybean lecithin (SL) (Lipoid S 75, with 70% of the phospholipids), and sodium oleate (Lipoid Sodium Oleate B) were purchased from Lipoid GmbH (Ludwigshafen, Germany). Ultra-pure water was used for the preparation of the formulations (GenPure apparatus, TKA Wasseranfbereitungssysteme GmbH, Neiderelbert, Germany). Other reagents were of pharmaceutical or HPLC grade and used without additional purification.

#### 2.1.2. Solubility

The solubility of GL-II-73 was investigated in various media using the shake flask method. In brief, GL-II-73 was added in excess and mixed overnight on the vortex, followed by centrifugation to remove any excess of undissolved drug. The supernatants were then diluted in methanol and GL-II-73 content was determined by the LC/MS-MS method.

#### 2.1.3. Development and Preparation of Nanoemulsions

The detailed composition of the developed nanoemulsions is shown in Table 1. The nanoemulsions were prepared using high-pressure homogenization.

In brief, the two phases were mixed separately and heated to 50 °C, until all components were dissolved. The aqueous phase was then added to the oil phase and mixed for 1 min at 11,000 rpm on a rotor–stator homogenizer (IKA Ultra-Turrax T25 digital, IKA-Werke GmbH & Co. KG, Staufen, Germany) to obtain pre-emulsions. The formulation was further processed on a high-pressure homogenizer (EmulsiFlex-C3, Avestin Inc., Ottawa, ON, Canada) for 10 discontinued cycles at 800 bar to obtain nanoemulsions. The optimal nanoemulsion formulation was selected and steam-sterilized in an autoclave at 121 °C for 15 min prior to in vitro and in vivo testing.

### 2.2. Physicochemical Characterization of Nanoemulsions

#### 2.2.1. Size Measurements

Droplet size (intensity weighted diameter, Z-ave) and droplet size distribution (polydispersity index, PDI) were analyzed using the dynamic light scattering (DLS) technique using a Zetasizer Nano ZS90 (Malvern Instruments Ltd., Worcestershire, UK) after dilution in highly purified water at a 1:500 (*v*/*v*) ratio.

#### 2.2.2. Zeta Potential

Before zeta potential measurements, nanoemulsions were diluted in highly purified water with a conductivity adjusted to 50 µS/cm with sodium chloride in the same ratio as for the size measurements. The analysis was performed in triplicate using a Zetasizer Nano ZS90 (Malvern Instruments Ltd., Worcestershire, UK).

#### 2.2.3. Conductivity and pH Values

The conductivity and pH of the developed formulations were measured by directly immersing the electrodes of the conductometer (sensIONTM + EC71, ShangHaiShilu Instruments Co., Ltd., Shanghai, China) and the pH meter (Hanna Instruments Inc., Ann Arbor, MI, USA) in the samples. The measurements were carried out in triplicate.

#### 2.2.4. GL-II-73 Content in NE

The content of GL-II-73 in nanoemulsions was determined after dilution of 25 µL of nanoemulsion in 50 mL isopropanol, and subsequent ultrasonic treatment in an ultrasonic bath for 15 min. The resulting solution was analyzed for GL-II-73 content using the LC/MS-MS technique.

#### 2.2.5. Encapsulation Efficacy (EE)

The encapsulation efficacy of GL-II-73 in nanoemulsions was determined using Amicon Ultra-4 NMWL 10 kDa filter units (Merck Millipore, Burlington, MA, USA) by adding 2 mL of the formulations to the tube and centrifuging at 3000 rpm for 45 min to separate the aqueous phase. The resulting filtrate (10 µL) was diluted with 1990 µL of isopropanol and analyzed for GL-II-73 content by LC-MS/MS. The encapsulation efficacy was then calculated with the help of the following equation (Equation (1)):

(1) $$\%EE=\frac{A_{formulation}-A_{filtrate}}{A_{formulation}}*100$$

where A_formulation_ signifies GL-II-73 content in the NE, and A_filtrate_ is the GL-II-73 content in the filtrate. These experiments were performed in duplicate.

#### 2.2.6. Analytical Method

A liquid chromatographic system, the Accela 1000 (Thermo Fisher Scientific, San Jose, CA, USA), which includes a quaternary pump and an autosampler, was used for chromatographic analyses. All runs were performed with an XTERRA^®^ MS C18 column (150 mm × 2.1 mm, 3.5 µm; Waters, Dublin, Ireland) maintained at 25 °C. Samples were stored in the autosampler temperature-controlled compartment at 8 °C. Isocratic elution was performed with a mixture of acetonitrile and 0.1% aqueous formic acid (60:40, *v*/*v*) at a flow rate of 300 µL/min. The total analysis time was 4.5 min. Mass spectrometric analyses were performed using a TSQ Quantum Access MAX (Thermo Fisher Scientific, Waltham, MA, USA) triple-quadrupole spectrometer equipped with a heated electrospray ionization (HESI) source. Operating conditions of the MS were optimized as follows: the spray voltage was set to 4000 V, with a skimmer offset of 0 V; the evaporator and capillary temperatures were set to 350 °C and 300 °C, respectively; nitrogen was used as both the nebulizer and auxiliary gas, with flow rates of 50 and 10 arbitrary units, respectively; and the collision gas pressure in q2 was maintained at 1.4 mTorr of argon. Data acquisition was performed with Selected Reaction Monitoring (SRM) in positive scan mode. The [M + H]^+^ transitions *m*/*z* of SRM were 387.2 → 342.2 (CE 21 V), 373.1 → 314.0 (CE 29 V), and 347.0 → 302.0 (CE 28 V) for GL-II-73, MP-III-022, and SHI-I-048A (as internal standard), respectively. The Thermo Xcalibur 2.1 software was used for data acquisition.

#### 2.2.7. Viscosity

The viscosity of the optimal nanoemulsion was measured with an air-bearing rheometer (Anton Paar, Graz, Austria), using a coaxial cylinder system (CC27 measuring bob with C-PTD 180/Air) with a shear rate range of 0.1–100 s^−1^ at 20 °C.

#### 2.2.8. Electron Paramagnetic Resonance (EPR) Spectroscopy

EPR spectroscopy was used to analyze the interfacial properties of the optimized formulation and the corresponding placebo to better understand the impact of the investigated ligand on the nanoemulsion’s stabilizing layer. This technique measures the absorption of microwaves by paramagnetic centers with unpaired electrons. For this study, two different amphiphilic probes labeled at different locations on the aliphatic chain, 5- and 16-doxylstearic acid (DSA), were used to provide information on different depths of the stabilization layer, closer to the aqueous surface and the oil phase for 5-DSA and 16-DSA, respectively.

EPR measurements were performed with an EMX EPR spectrometer (Bruker BioSpin GmbH, Rheinstetten, Germany) in X-band (9.8 GHz) with a flat aqueous quartz sample cell (Wilmad-LabGlass, Cortecnet Europe, Voisins-Le-Bretonneux, France). The instrument settings were as follows: receiver gain 5.64 × 10^4^, scan range 0.01 T, central field 0.348 T, time constant 10.24 ms, conversion time 5 ms, and modulation amplitude 0.4 mT. For the analysis, 15 μL of the spin probe stock solution (1 mM) prepared in absolute ethanol was added to Eppendorf tubes and left to evaporate until completely dry. Subsequently, 1 mL of the nanoemulsions was added and allowed to stand at room temperature for 24 h to allow for the spin probe’s solubilization in the surfactant layer, resulting in the final concentration of the spin probes of 0.015 mM.

The Bruker WinEPR acquisition and processing program (Bruker BioSpin GmbH, Ettlingen, Germany) was used for data acquisition and analysis. The obtained spectra were analyzed to calculate the rotational correlation time (τR), the order parameter (S), and the isotropic hyperfine coupling constant (αN) according to previously published equations [16,17].

### 2.3. Blood–Brain Barrier (BBB) In Vitro Model

#### 2.3.1. Materials Used in the In Vitro Experiment

The iCell^®^ Blood-Brain Barrier Kit (FUJIFILM Cellular Dynamics, Inc., Madison, WI, USA) contained the following: iCell Astrocytes, iCell BMEC (Brain Microvascular Enothelial Cells), iCell Pericytes, iCell Astrocytes & iCell Pericytes Medium, iCell BMEC Maintenance Medium, and iCell Plating Supplement 500×. Additionally, in the development of the in vitro model of the blood–brain barrier, 24-well plates with BioCoat^®^ Control cell culture inserts, a PET membrane (Corning^®^ Incorporated, Salt Lake City, UT, USA), Collagen IV (Sigma-Aldrich, Burlington, MA, USA), Dulbecco’s phosphate-buffered saline without Ca⁺⁺ and Mg⁺⁺ (DPBS) (Gibco, Thermo Fisher Scientific, Waltham, MA, USA), Fibronectin (Sigma-Aldrich, Burlington, MA, USA), 0.1% gelatin in water (STEMCELL Technologies, Vancouver, BC, Canada) and Penicillin–Streptomycin (Gibco, Thermo Fisher Scientific, Waltham, MA, USA) were used.

GL-II-73 is a ligand with moderate water solubility, so a 90 μM solution of GL-II-73 was prepared with ultrapure water, diluted with iCell Maintenance Medium to a concentration of 3 μM, and then the indicated volume (300 μL) was applied to the apical side of the insert.

#### 2.3.2. Protocol for the Formation of the In Vitro BBB and TEER Measurement

The iCell^®^ Blood-Brain Barrier Kit (FUJIFILM Cellular Dynamics, Madison, WI, USA) contained astrocytes, pericytes, and brain microvascular endothelial cells. This model was used to investigate the BBB permeation of GL-II-73 contained in nanoemulsion. For comparison, the contribution of the carrier itself was evaluated using a GL-II-73 solution in ultrapure water. The protocol for the BBB experiment can be found in Supplementary Table S1, while a more detailed version is provided by the kit manufacturer [18].

The transendothelial electrical resistance (TEER) parameter was used to verify the integrity of the barrier. TEER measurements were performed using an EVOM2 Epithelial Ohm Meter equipped with chopsticks (World Precision Instruments, Sarasota, FL, USA). The measured TEER values were corrected with TEER values from wells with empty filters and the medium. The final TEER values (Ω × cm^2^) were calculated by adjusting the corrected TEER values to the filter surface. Once the integrity of the barrier was confirmed, 300 µL of a 3 µM dilution of the test formulations (nanoemulsion or solution) in the iCell Maintenance Medium was applied to the cells and the penetration of GL-II-73 was tested at predetermined time intervals. For this purpose, 100 µL was taken from the basolateral side (containing 1 mL iCell Maintenance Medium) to be analyzed by LC/MS-MS. To maintain the sink conditions, 100 µL of fresh iCell Maintenance Medium was added to the basolateral compartment. The possible binding of GL-II-73 to the plastic material of which the insert filter is made was also investigated to rule out any effects on the penetration results obtained by applying the formulations to the non-cell-loaded inserts.

#### 2.3.3. Calculation of Parameters

The Pe parameter (permeability coefficient; cm/min) was calculated as described earlier [19,20] and used to evaluate the rate and the effectiveness of GL-II-73 transport through the BBB. Firstly, the cleared volume (CL in μL) at each time point was determined by dividing the amount of substance in the receiver (basolateral) compartment (A_receiver_) by the drug concentration in the donor (apical) compartment (C_donor_) (Equation (2)).

(2) $$CL=\frac{A_{receiver}}{C_{donor}}$$

When the average CL at each time point is plotted over time, the slope corresponds to the permeability surface area product (PS, in μL/min) of the filter. The PS of an insert coated with BBB cells is referred to as the total PS (PSt) and the PS of an insert without BBB cells is referred to as the filter PS (PSf). The PS value of the GL-II-73 for the BBB-Tri culture (Pse) was calculated from PSt and PSf (Equation (3)).

(3) $$\frac{1}{PSe}=\frac{1}{PSt}-\frac{1}{PSf}$$

The Pe value was determined by dividing the Pse values by the surface area of the membrane of the insert (cm^2^). Mass balance (MB, in %) was used to evaluate the adsorption of GL-II-73 to plastic or non-specific binding to cells by dividing the total amount of compound recovered in both compartments at the end of the experiment by the initial amount of compound introduced into the donor compartment (apical) at t_0_.

### 2.4. In Vivo Neuropharmacokinetic Study

#### 2.4.1. Materials and Animals for the Neuropharmacokinetic Study

The neuropharmacokinetic study was performed after intraperitoneal administration of the optimized nanoemulsion (NE4) or a 2 mg/mL GL-II-73 solution (solvent consisting of 14% (*w*/*v*) propylene glycol and 1% (*w*/*v*) polysorbate 80 in ultrapure water). Female Sprague Dawley rats, approximately two months old and weighing 160–200 g, were housed four per cage. The animal room was kept at a temperature of 22 ± 1 °C and a relative humidity of 40–70%. The alternation of light and darkness took place in a 12 h rhythm (light at 6:00 a.m.) with an illumination of 120 lx. The research was conducted in accordance with the guidelines of the National Institutes of Health Animal Care and Use Committee. Approval was granted by the Ethics Committee for Animal Experiments of the University of Belgrade—Faculty of Pharmacy, Serbia, and the Ministry of Agriculture, Forestry and Water Management—Veterinary Directorate (323-07-10046/2020-05).

#### 2.4.2. Experimental Design

The test animals were divided into two groups of 27 rats each (3 animals per time point). One group received the GL-II-73 solution, the other group the GL-II-73 nanoemulsion, each at a dose of 10 mg/kg. At predefined intervals (5 min, 15 min, 30 min, 1 h, 2 h, 4 h, 8 h, 16 h, and 36 h), the animals were anesthetized with ketamine hydrochloride 90 mg/kg (Ketamidor, Richter Pharma AG, Vienna, Austria) and blood and brain samples were taken. Blood was collected by cardiac puncture with heparinized syringes and centrifuged at 1000× *g* for 10 min (MiniSpin^®^ plus centrifuge, Eppendorf, Hamburg, Germany) to separate plasma. The isolated brains were weighed and homogenized in 1 mL methanol using an ultrasonic probe. The homogenized brain samples were subsequently centrifuged at 3400× *g* for 20 min to collect the supernatants. Plasma and the supernatants of the brain homogenates were further purified by solid phase extraction using Oasis HLB cartridges (Waters Corporation, Milford, MA, USA). The cartridges were preconditioned with methanol and then with water prior to application of the sample and internal standard. Endogenous impurities were removed by washing the cartridges with water and methanol, while elution was performed with 1 mL of methanol for 1 min. The concentration of GL-II-73 and its metabolite MP-III-022 was quantified in the eluates by liquid chromatography–tandem mass spectrometry (LC-MS/MS). Certara Phoenix WinNonlin™ software v.8.5 was used to process the results, which include the pharmacokinetic parameters C_max_ (maximum concentration), T_max_ (time to reach maximum concentration), AUC_0-t_ (area under the concentration curve as a function of time from zero to the last measurement point), and t_1/2_ (elimination half-life). The concentration was quantified using an analytical method as described in Section 2.2.6.

### 2.5. Statistical Analysis

Statistical analysis was performed using GraphPad Prism (version 10.1.0; GraphPad Software, Inc., La Jolla, CA, USA). The differences in physicochemical parameters measured upon preparation and after storage, as well as in the parameters determined in the in vivo pharmacokinetic experiment, were studied using the independent Student’s *t*-test. *p* values less than 0.05 were considered statistically significant, with *p* < 0.01 and *p* < 0.001 indicating highly significant and very highly significant results, respectively. It is important to note that borderline significant results (e.g., *p* values close to 0.05) should be interpreted with caution. Such findings were further evaluated in the context of effect sizes and their practical or clinical relevance to avoid overinterpretation.

## 3. Results and Discussion

### 3.1. Physicochemical Characterization of Nanoemulsions

The solubility of GL-II-73 in the investigated media is shown in Table 2. GL-II-73 exhibits relatively good oil solubility, which makes nanoemulsions promising delivery systems for its future parenteral administration. The solubility of GL-II-73 in benzyl alcohol, a lipophilic cosolvent previously used to achieve increased drug loading in nanoemulsions [21,22] was deemed to be excellent, but due to its adequate solubility in oils it was not needed for further research. The best solubility was observed in MCT, making it the oil phase of choice for nanoemulsion preparation. In addition, nanoemulsions were prepared with soybean oil (NE3) to investigate the effects of this oleic acid-rich oil on their stability, as it has been reported to have a stabilizing effect on nanoemulsions [16,23]. From the results of solubility in phosphate buffer 7.4 and 0.1 M HCl (Table 2), it was concluded that GL-II-73 has a pH-dependent solubility. These results could probably be due to the presence of ionizable functional groups and several H-bond acceptors in the GL-II-73 structure (Figure 1). In this context, we hypothesized that the best encapsulation could be achieved by increasing the pH of the aqueous phase, which was achieved by adjusting the pH of the aqueous phase with sodium oleate, which acts as an additional costabilizer (NE1), or with the pH 8 phosphate buffer (NE2 and NE3). The higher solubility in methanol compared to isopropanol (Table 2) could be due to the higher polarity index, which is why methanol is the solvent of choice whenever possible.

Table 3 shows that the formulations NE1-NE3 exhibited properties suitable for parenteral administration, with a small droplet size (<500 nm), narrow droplet size distribution (<0.25), and absolute zeta potential values above 30 mV [23,24]. Although the initial pH values were acceptable for parenteral administration (3.5 ≤ pH ≤ 9) [25], they dropped significantly during storage (*p* < 0.001), indicating formulation instability, implying the inadequacy of sodium oleate as a costabilizer in the case of NE1. For NE2 and NE3, the decrease in pH values was also significant (*p* < 0.001), but not as profound as for NE1. However, these nanoemulsions disintegrated during heat sterilization, suggesting that the ionic strength of the buffer had a detrimental effect on the stability of the formulation (Supplementary Figure S1), so they were excluded from further investigation.

The final formulation studied, NE4, contained only highly purified water with no other pH-adjusting ingredients. Due to the preliminary characterization, especially the lower pH drop and the good stability during autoclaving, this nanoemulsion was selected as optimal for further investigations and its stability was followed over a longer period (one year). The analyzed physicochemical parameters of the autoclaved NE4 formulation, both after preparation and after one year of storage, are shown in Table 4. Similar to the other formulations (NE1-3), the Z-ave, PDI, and ZP values were suitable for parenteral administration [23,24] and remained stable during the study period. Interestingly, the pH and conductivity did not change drastically compared to the NE1-3 samples, which was particularly important as the stability of these formulations was only monitored for one month, whereas NE4 was analyzed after one year of storage. Viscosity values of 6.9435 mPa*s indicate good injectability [21]. The content of GL-II-73 changed slightly during storage, which could be a consequence of the different droplet sizes in the sample, which could not be detected with the DLS measurement, due to its limitations. The drop in GL-II-73 content was not considered drastic, particularly given the time lapse between the two measurements, and was not taken as a sign of formulation instability. It should also be noted that the encapsulation efficacy was high, at 97.26 ± 0.06 and 96.79 ± 0.01 at the beginning and after one year, respectively, indicating a high inclusion of GL-II-73 in the oil droplet. The initial physicochemical assessment of NE4 and the corresponding placebo revealed similar parameter values. An additional EPR study was conducted to better understand the impact of GL-II-73 on the stabilizing layer of the nanoemulsion.

**Figure 2:**
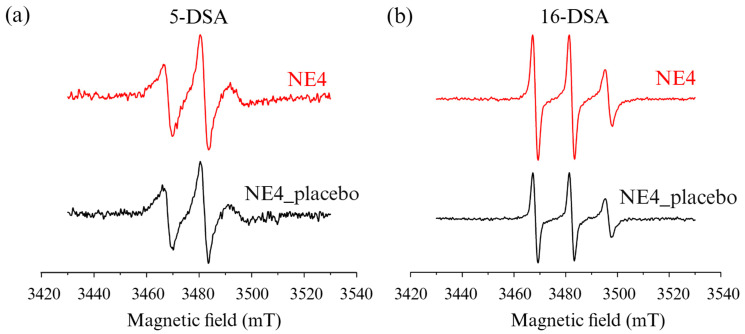
EPR spectra of the optimized NE4 nanoemulsion in (**a**) 5-DSA and (**b**) 16-DSA spin probes.

EPR spectroscopy was used to determine the exact localization of GL-II-73 within the droplet. The spectra of the 5-DSA and the 16-DSA probes for NE4 and the corresponding placebo with similar physicochemical properties (Table 4) are shown in Figure 2.

The calculated values for the EPR parameters (Table 5) revealed that the greatest difference between the ligand-loaded and placebo formulations for the 5-DSA probe was observed in the τR values. Higher τR values in the GL-II-73-loaded nanoemulsions indicate not only that GL-II-73 is present in the stabilizing layer, in the part closer to the aqueous phase, but also that its addition increases the stiffness of the stabilizing layer, potentially improving the physicochemical stability of the nanoemulsion. Similar results have been obtained with a curcumin-loaded nanoemulsion, where the addition of curcumin improved the long-term stability of parenteral nanoemulsion with fish oil [21]. The values of the other parameters, S and αN, did not change with the addition of GL-II-73, indicating that local polarity and isotropy were not significantly affected. On the other hand, the τR values were lower for the 16-DSA probe, reflecting a higher mobility of the stabilizing layer in the part closest to the oil phase. The addition of GL-II-73 did not cause significant changes in the values of spectral parameters for the 16-DSA probe, indicating that GL-II-73 does not penetrate deeper into the interior of the droplet.

### 3.2. In Vitro Permeability Test of the BBB Model

The BBB has a peculiar architecture, with its tightly interconnected endothelial capillary cells surrounded by pericytes, the basal lamina, and astrocytic perivascular endfeet, and plays a crucial role in maintaining brain homeostasis [26]. It serves as the primary interface between the circulatory system and the brain and often impedes drug access to these important targets [20].

Most current BBB models hardly take into account the complexity of the BBB, which prevents a comprehensive understanding of its functions [27]. The tricellular BBB model, which consists of human induced pluripotent stem cell-derived brain microvascular endothelial cells, astrocytes, and pericytes, provides a more physiologically relevant representation of the BBB compared to monolayer models. The inclusion of astrocytes and pericytes improves barrier integrity by promoting higher TEER, greater expression of tight junction proteins [26], and functionally polarized transport, which closely resemble the physiology of the human BBB [28]. However, the in vitro model of the BBB has some limitations, such as the following: fluid flow could not be taken into account; the influence of the basal lamina is not considered; it is not able to mimic the cylindrical geometry in vivo; and it cannot fully reproduce the complexity of the human BBB in vivo, especially with regard to the active transport mechanisms. Some of the limitations could be overcome by more complex models, such as organ-on-chip systems [29]. The iCell^®^ Blood-Brain Barrier Isogenic Kit was selected to study the transport of our formulations as it best mimics the physiological properties of the human BBB in vitro. This kit enables the co-culture of isogenic human brain microvascular endothelial cells, astrocytes, and pericytes, ensuring genetic consistency and reducing variability in experimental results. The system exhibits robust barrier integrity, as evidenced by high TEER values and tight junction formation, and supports complex cell–cell interactions that are critical for accurate modeling of BBB function. Its scalability and compatibility with high-throughput applications also make it an ideal choice for drug permeability testing and targeted CNS therapies [30].

The calculated TEER values (Supplementary Table S2) indicate an intact membrane. Values around 4000 Ω*cm^2^ reported for other in vitro BBB models, such as co-cultures of primary human brain pericytes, human astrocytes, and neurons derived from human neural progenitor cells, were in agreement with the values obtained in our study [18,31,32]. TEER was used as a reliable indicator of barrier integrity prior to evaluating drug or chemical transport and was therefore only measured on the third and fourth day of the BBB experiment (Supplementary Table S1) so as not to disturb the established gradient or membrane permeability once the formulations were applied.

The Pe value for the optimal nanoemulsion (NE4) (6.3 × 10^−3^ cm/min) was more than twice that of the solution (2.99 × 10^−3^ cm/min) (Table 6), supporting the hypothesis that the nanoemulsion enhanced the transport of GL-II-73 through the BBB. This difference could be attributed to the increased lipophilicity of the nanoemulsion, which facilitates its integration into and passage through the lipophilic cell membranes of the BBB [4]. On the other hand, it was observed that the calculated PSt values were similar for both the nanoemulsion and the solution, but there was a difference in the PSf parameter. This could be a consequence of the higher viscosity of the nanoemulsion (6.9435 mPa×s) compared to the solution, which slows down the transport of GL-II-73 through the insert membrane (filter). The Pe results obtained were particularly interesting as the GL-II-73 was located in the stabilizing layer of the nanoemulsion, implying that its localization could not only improve the physicochemical properties of the formulation but also affect its performance under biologically relevant conditions. Moreover, the Pe values obtained were similar to those of diazepam (4.20 × 10^−3^ cm/min), which was expected given the structural similarity [20]. The calculated MB values were similar for the nanoemulsion and the solution: 126.64 ± 9.68% and 121.47 ± 21.59%, respectively (Table 6). Reported optimal MB values, indicating accurate measurement and minimal loss due to degradation or adsorption, are generally between 80% and 120% [33,34], which is consistent with our results (Table 6).

### 3.3. In Vivo Neuropharmacokinetic Study

**Figure 3:**
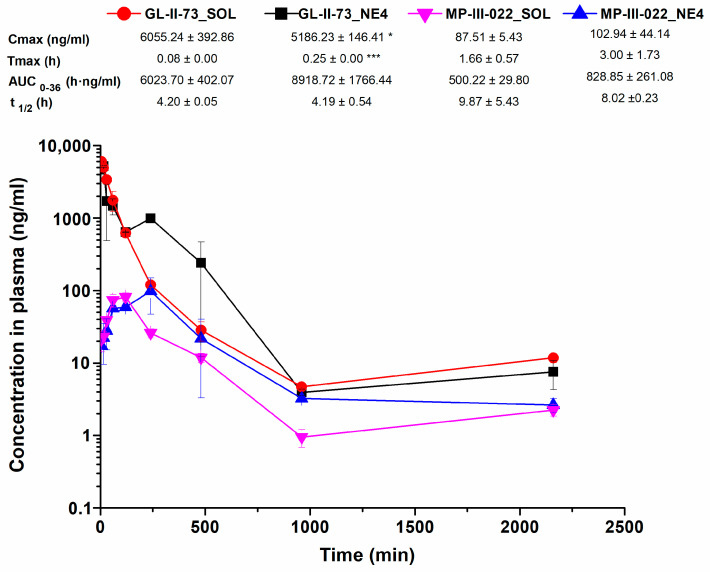
Plasma concentration–time profile of GL-II-73 and MP-III-022 and calculated pharmacokinetic parameters after intraperitoneal administration of 10 mg/kg dose of GL-II-73 nanoemulsion or solution (*n* = 3 per time point). (C_max_ = maximum concentration in plasma; T_max_ = time of maximum concentration in plasma; t_1/2_ = terminal elimination half-life from plasma; AUC_0-36_ = area under the plasma concentration–time curve from 0 to 36 h; means ± SD, *n* = 3). * and ***, *p* < 0.05 and *p* < 0.001.

**Figure 4:**
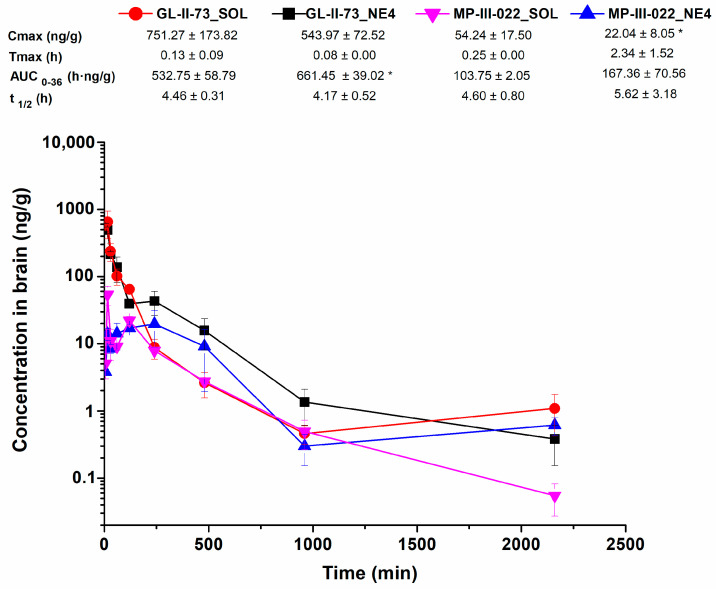
Brain concentration–time profile of GL-II-73 and MP-III-022 and calculated pharmacokinetic parameters after intraperitoneal administration of 10 mg/kg dose of GL-II-73 nanoemulsion or solution (*n* = 3 per time point). (C_max_ = maximum concentration in brain; Tmax = time of maximum concentration in brain; t_1/2_ = elimination half-life from brain; AUC_0-36_ = area under the brain concentration–time curve from 0 to 36 h; means ± SD, *n* = 3). *, *p* < 0.05.

Concentration–time curves for plasma and brain, for the nanoemulsion and the corresponding solution and both the parent molecule GL-II-73 and its metabolite MP-III-022, are presented in Figure 3 and Figure 4, respectively.

GL-II-73 reached a lower maximum plasma concentration in rats that were administered with nanoemulsion rather than the solution (*p* = 0.023). In addition, the maximum concentration was reached later when the nanoemulsion was administered (Figure 3). These results could be explained by the formation of temporary GL-II-73 depots at the injection site, which slow down the release of GL-II-73 into the blood (cf. [22]). In contrast, the AUC_0–36_ values for GL-II-73 from the nanoemulsion tended to be higher compared to the solution, although the difference was of borderline significance (*p* = 0.050), which is likely due to inter-individual variation. This suggests that lower maximum concentrations but higher systemic exposure could be achieved with nanoemulsion treatment, which is consistent with previously published results for nanoemulsions containing drugs such as cefuroxime and valproic acid [23,35]. The elimination half-life (t_1/2_) of GL-II-73 was similar for both treatments.

With respect to MP-III-022, there were no significant differences in the pharmacokinetic profiles and parameters calculated after administration of the nanoemulsion or the solution. Nevertheless, a higher AUC_0–36_ for MP-III-022 tended to be achieved with the nanoemulsion, but the difference was not statistically significant (*p* = 0.096). This result is consistent with the plasma exposure data of the parent molecule and clearly shows that the nanoemulsion provides good systemic availability.

For GL-II-73, there were no significant differences in the neuropharmacokinetic profiles and parameters in brain tissue calculated after administration of the nanoemulsion or the solution, with one important exception (Figure 4). The AUC_0–36_ value was significantly higher after the administration of the nanoemulsion compared to the solution (*p* = 0.034), indicating that a higher total tissue exposure to the parent molecule was achieved. This result correlates with the Pe values obtained in our in vitro BBB permeability assay. Interestingly, in the study with the valproic acid nanoemulsions, no similar correlation was observed between the Pe values in the in vitro BBB permeability test and the AUC in the in vivo pharmacokinetic experiment, possibly due to the different composition of the BBB [35]. Nanoemulsion formulations have also obtained an improved brain delivery of cefuroxime, risperidone, and carbamazepine [10,22,23].

In vitro BBB models are often validated by comparing Pe values with in vivo permeability data. The strong correlation between in vitro and in vivo permeability underscores the utility of Pe as a reliable indicator for predicting brain exposure to CNS-targeted drugs [20]. This was demonstrated in a study [36] that showed a strong correlation between in vitro Pe values from a BBB model and in vivo BBB permeability data. In another study [37], the correlation between the in vitro and in vivo permeability of drugs in the brain was investigated, focusing on the predictive value of the iPSC–human BBB model (iPSC-hBBB). A significant correlation was found between the in vitro permeability coefficients and in vivo pharmacokinetic data derived from PET imaging, a technique used to assess drug exposure in the human brain. The current study focused on one rather than multiple compounds, and the correlation between the in vitro Pe values obtained from measurements on the BBB model and the in vivo BBB permeability data [36] cannot be established. Nevertheless, the higher in vitro Pe value for GL-II-73 in the nanoformulation compared to the solution was translated into an increased brain exposure, i.e., a one-third higher sum of AUC_0-36_ values for the parent molecule and its metabolite after systemic administration of the respective formulations of GL-II-73. Although further validation with a larger data set is required, our results suggest that the iPSC-hBBB model used is a valuable tool for assessing the brain penetration of drug candidates. Ultimately, it could be integrated into drug development pipelines for the treatment of brain diseases, with miniaturization and high-throughput screening strategies planned for the future.

The metabolite MP-III-022 was not quantified in the in vitro model of the BBB, as it is assumed to be synthesized in the liver (e.g., ref. [38]) and not in the microvascular endothelial and glial cells. In the neuropharmacokinetic study, the maximum concentration of MP-III-022 in the brain was lower after administration of the nanoemulsion than after administration of the solution (*p* = 0.044). In addition, it took longer for MP-III-022 to reach Tmax after administration of the nanoemulsion compared to the solution (Figure 3). On the other hand, the AUC_0–36_ value of MP-III-022 was 61% higher in the nanoemulsion compared to the solution, although this difference was not statistically significant (*p* = 0.194), apparently due to considerable interindividual variability. These results could possibly be explained in part by the fact that MP-III-022 is a metabolite and therefore took longer to be formed, especially in the nanoemulsion, as GL-II-73 first had to be released from the oil droplet and then metabolized.

## 4. Conclusions

GL-II-73, a positive allosteric modulator selective for α5GABAA receptors, exhibited more than twofold higher permeability in the human cell-based BBB model when released from the optimized nanoemulsion rather than from the solution. The increased permeability in vitro is at least partly due to its localization in the stabilizing layer of the nanoemulsion and proved to be a good predictor for the improved in vivo availability of GL-II-73 in brain tissue from the formulation obtained by encapsulation in a nanoemulsion. The improved exposure profile in the brain could lead to a reduction in GL-II-73 doses in future animal studies and potential parenteral therapeutic applications, with the potential to reduce dosing frequency and increase safety, including reduced risk of off-target and/or non-CNS adverse effects. Considering that a potential clinical application includes neuropsychiatric conditions that may be characterized by urgency and/or the need for prolonged precise dosing in hospitalized patients, such as affective and cognitive deficits in major depression [39], the nanoemulsion formulation may provide a means to optimize patient outcomes prior to potential transferring to oral GL-II-73. In addition, the neuropharmacokinetic study showed that systemic administration of GL-II-73 results in measurable concentrations of its mono-demethyl metabolite MP-III-022, which was also postulated to be a positive allosteric modulator selective for α5GABA_A_ receptors. Although the difference is not statistically significant due to interindividual variability, the active metabolite was also substantially more present in brain tissue after administration of the selected nanoemulsion compared to the solvent. The additive effect of the parent molecule and its metabolite may lead to enhanced and/or prolonged modulation of α5GABA_A_ receptors.

## Figures and Tables

**Table 1:** Composition of investigated nanoemulsions.

Ingredients (%, *w*/*w*)	NE1	NE2	NE3	NE4
Oil phase				
Soybean oil	/	/	4	
MCT	20	20	16	20
SL	2	2	2	2
BHT	0.05	0.05	0.05	0.05
Aqueous phase				
Glycerol	2.25	2.25	2.25	2.25
Polysorbate 80	2	2	2	2
GL-II-73	0.2	0.2	0.2	0.2
Sodium oleate	0.03	/	/	/
Phosphate buffer pH 8; 0.01 M	/	to 100	to 100	/
Water	to 100	/	/	to 100

MCT: medium-chain triglyceride, SL: soybean lecithin; BHT: buthylhidroxy toluen.

**Table 2:** GL-II-73 solubility in selected solvents.

Solvent	Solubility (µg/mL)
Water (pH 5.2)	1001.10 ± 39.94
0.1 M HCl (pH 1.2)	5370.70 ± 195.26
Phosphate buffer (pH 7.4)	951.37 ± 41.38
MCT	4489.70 ± 148.32
Soybean oil	3055.05 ± 137.42
Castor oil	2820.65 ± 183.68
Fish oil	2395.07 ± 331.00
Benzyl alcohol	>534,365.79 ± 80,924.95
Isopropanol	131,047.81 ± 6902.35
Methanol	>1,469,735.25 ± 93,891.20

The values are presented as means ± sd.

**Table 3:** Physicochemical stability of NE1-NE3 formulations.

Parameters	NE Formulations			
		NE1	NE2	NE3
Z-ave (nm)	In	115.0 ± 1.9	115.1 ± 1.7	120.3 ± 0.3
	1 m	119.1 ± 1.3 *	116.4 ± 1.5	122.0 ± 0.8 *
PDI	In	0.136 ± 0.015	0.099 ± 0.012	0.077 ± 0.005
	1 m	0.070 ± 0.016 **	0.082 ± 0.032	0.063 ± 0.017
ZP (mV)	In	−47.7 ± 1.7	−43.4 ± 0.4	−44.1 ± 1.3
	1 m	−45.0 ± 1.3	−44.5 ± 1.2	−44.0 ± 0.8
pH	In	7.75 ± 0.02	7.82 ± 0.02	7.84 ± 0.01
	1 m	5.13 ± 0.03 ***	6.99 ± 0.01 ***	6.75 ± 0.03 ***
Conductivity (µS/cm)	In	159.37 ± 3.52	1073.67 ± 1.53	1057 ± 4.36
	1 m	401.33 ± 1.53 ***	1025.00 ± 4.58 ***	1004.33 ± 1.53 ***

The values are presented as means ± sd (*n* = 3); *, **, and ***, *p* < 0.05, *p* < 0.01, and *p* < 0.001 compared to the initially measured values.

**Table 4:** Physicochemical properties of the optimized formulation (NE4) and corresponding placebo, measured initially (In) and after 1 year (1 y).

Parameters		NE4	NE4_placebo
Z-ave (nm)	In	122.0 ± 1.5	117.9 ± 0.5
	1 y	124.9 ± 1.2	n.d.
PDI	In	0.123 ± 0.009	0.09 ± 0.01
	1 y	0.094 ± 0.020	n.d.
ZP (mV)	In	−40.7 ± 1.5	−39.0 ± 0.4
	1 y	−40.0 ± 0.4	n.d.
pH	In	5.16 ± 0.04	5.53 ± 0.02
	1 y	4.80 ± 0.01 ***	n.d.
Conductivity (µS/cm)	In	128.03 ± 1.29	88.83 ± 0.49
	1 y	120.40 ± 1.25 **	n.d.
GL-II-73 content (mg/mL)	In	2.32 ± 0.07	n.d.
	1 y	1.93 ± 0.04 **	n.d.

The values are presented as means ± sd (*n* = 3). ** and ***, *p* < 0.01 and *p* < 0.001 compared to the initially measured values. n.d.—not determined.

**Table 5:** Calculated values of the EPR spectra parameters.

	NE4_placebo		NE4	
	5-DSA	16-DSA	5-DSA	16-DSA
τR (ns)	2.80 ± 0.01	0.60 ± 0.03	2.93 ± 0.07	0.58 ± 0.02
S	0.18 ± 0.04	0.03 ± 0.00	0.17 ± 0.01	0.05 ± 0.00
αN (×10^−4^ T)	13.36 ± 0.26	14.66 ± 0.05	13.25 ± 0.40	14.57 ± 0.06

τR—rotational correlation time; S—order parameter; αN—isotropic hyperfine coupling constant.

**Table 6:** Calculated parameters for in vitro BBB permeability test.

Parameters	Formulations	
	Solution	Nanoemulsion
PSt	0.3317	0.3215
PSf	0.4989	0.3803
PSe	0.9897	2.0794
Pe (×10^−3^ cm/min)	2.99	6.30
MB (%) *	121.47 ± 21.59	126.64 ± 9.68

PSt—total PS; PSf—filter PS (PSf). Pse—PS value for the BBB model; MB–mass balance. * values are shown as means ± SD.

## Data Availability

All raw data will be made available upon the request to the corresponding author.

## Supplementary Materials

The following supporting information can be downloaded at https://www.mdpi.com/article/10.3390/pharmaceutics17030354/s1: Figure S1: (a) NE1 and NE2 formulations with corresponding placebos after autoclaving; (b) NE3 and corresponding placebo after autoclaving; Table S1: Protocol for BBB model experiments; Table S2: Transendothelial electrical resistance (TEER) (Ω*cm^2^).

## Abbreviations

The following abbreviations are used in this manuscript:

BBB	Blood–Brain Barrier
BHT	Butylhydroxyltoluene
BMEC	Brain Microvascular Endothelial cells
CNS	Central Nervous System
DLS	Dynamic Light Scattering
DMSO	Dimethyl Sulfoxide
EE	Encapsulation Efficiency
EPR	Electron paramagnetic resonance
GABA_A_	γ-aminobutyric acid type A
HESI	Heated electrospray ionization
HPLC	High-performance liquid chromatography
HLB	Hydrophilic–Lipophilic Balance
iPCS	Induced Pluripotent Stem Cells
LC-MS/MS	Liquid Chromatography with Tandem Mass Spectrometry
MB	Mass balance
MCT	Medium Chain Triglycerides
NE	Nanoemulsion
PBS	Phosphate-Buffered Saline
PDI	Polydispersity Index
Pe	Permeability Coefficient
PET	Positron emission tomography
PS	Permeability surface area product
ISD	Standard Deviation
SL	Soybean lecithin
SOL	Solution
SRM	Selected Reaction Monitoring
TEER	Trans Endothelial Electrical Resistance
UHPLC	Ultra-High-Performance Liquid Chromatography
Z-ave	Intensity Weighted Mean Hydrodynamic diameter
ZP	Zeta Potential
α5GABA_A_ receptors	GABA_A_ receptors containing the α5 subunit
