# Supplementary material for: Parenteral Nanoemulsion for Optimized Delivery of GL-II-73 to the Brain—Comparative In Vitro Blood–Brain Barrier and In Vivo Neuropharmacokinetic Evaluation
Source: Pharmaceutics. 2025 Mar 10;17(3):354. doi: 10.3390/pharmaceutics17030354 (PMC11944986; doi:10.3390/pharmaceutics17030354)
Supplement: Supplementary file 1 [file pharmaceutics-17-00354-s001.zip › pharmaceutics-3431461-supplementary.pdf]

Supplementary material

Parenteral nanoemulsion for optimized delivery of GL-II-73 to the brain – comparative in vitro blood-brain barrier and in vivo neuropharmacokinetic evaluation

Kristina Jezdić[1], Jelena Đoković[2], Ivan Jančić[3*], Tanja Ilić[2], Biljana Bufan[3], Bojan Marković[4], Jana Ivanović[1], Tijana Stanković[2], Nebojša D. Cekić[5], Vassiliki Papadimitriou[6], Dishary Sharmin[7], Prithu Mondal[7], James M. Cook[7], Snežana D. Savić[2], Miroslav M. Savić[1]

[1]Department of Pharmacology, University of Belgrade – Faculty of Pharmacy, Belgrade, Serbia

[2]Department of Pharmaceutical Technology and Cosmetology, University of Belgrade – Faculty of Pharmacy, Belgrade, Serbia

[3]Department of Microbiology and Immunology, University of Belgrade – Faculty of Pharmacy, Belgrade, Serbia

[4]Department of Pharmaceutical Chemistry, University of Belgrade – Faculty of Pharmacy, Belgrade, Serbia

[5]Faculty of Technology, University of Niš, Leskovac, Serbia; DCP Hemigal, Leskovac, Serbia

[6]Institute of Chemical Biology, National Hellenic Research Foundation, Athens, Greece

[7]Department of Chemistry and Biochemistry, Milwaukee Institute for Drug Discovery, University of Wisconsin-Milwaukee, United States

a) b)

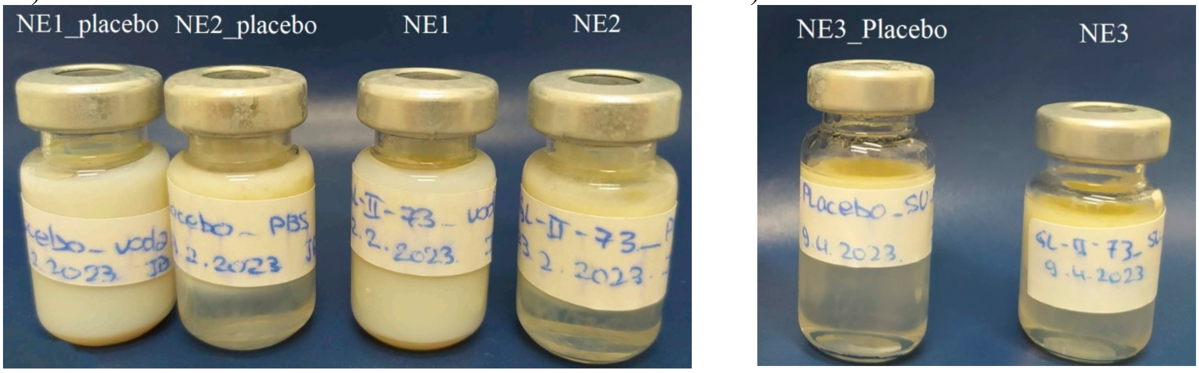

Figure S1. a) NE1 and NE2 formulations with corresponding placebos after autoclaving; b) NE3 and corresponding placebo after autoclaving

Supplementary Table S1. Protocol for BBB model experiments

Day of model preparation	Activities
Day - 2	-Coating of cell culture inserts with a fibronectin-collagen IV matrix on the apical (A) side
Day - 1	**-Preparation of the iCell Plating Medium**<br>-Coating **the basolateral (BL) side of the cell culture inserts with a 0.1% gelatin solution**<br>**-Seeding of iCell Astrocytes and iCell Pericytes** onto the basolateral membrane of the cell culture inserts, followed by a 4-hour incubation and addition of the medium (1 mL) to the basolateral side<br>-Removal of the fibronectin-collagen IV solution from the apical side and addition of the iCell AP Plating Medium (300 µL)
Day 0	**-Preparation of the maintenance medium**<br>-Asspiration of the iCell AP Plating Medium from the apical and basolateral compartments and addition of 1 mL of iCell BMEC Plating Medium to the basolateral compartment of each seeded cell culture insert<br>-Addition of 300 µL of the **iCell BMEC cell suspension to each apical compartment and subsequent incubation overnight**
Day 1*	-Replacing the iCell BMEC Plating Medium with the iCell BMEC Maintenance Medium on both the apical and basolateral compartments<br>-Formation of the membrane
Day 3	-Measuring TEER values<br>-Refreshing the iCell BMEC Maintenance Medium (removing the existing medium and adding fresh one)
Day 4	-Measuring TEER values<br>-Refreshing the iCell BMEC Maintenance Medium (removing the existing medium and adding fresh one)<br>-Application of the NE and solution loaded with GL-II-73<br>-Sampling the contents of the cell wells 2, 6 and 24 hours after treatment on the basolateral side and after 24 hours on the apical side

* Formation of the membrane; days -2 – 0: preparation stage

Table S2. Transendothelial electrical resistance (TEER) (Ω*cm^2)

Wells*	TEER, day 3 (Ω*cm^2)	TEER, day 4 (Ω*cm^2)
1	1373	3333
2	1457	2574
3	1023	2227
4	1531	3498
5	1254	2326
6	1402	3102

*well number 1, 2 and 3 were used for NE, while 4, 5 and 6 were used for solution
